# Romania – experience and new steps in the context of the international patient classification system

**DOI:** 10.1186/1472-6963-11-S1-A12

**Published:** 2011-10-19

**Authors:** ND Chiriac, SN Musat, J Shah, C Vladescu

**Affiliations:** 1 Center for Health Research and Evaluation, National School of Public Health, Management and Professional Development in Health, Bucharest, 021253, Romania; 2 Nimitt Consulting Inc., 2038 18th Street NW, Washington DC, 20009, USA; 3 National School of Public Health, Management and Professional Development in Health Bucharest, 021253, Romania

## Introduction

Over the last 10 years many countries, including Romania, have introduced various models of Casemix financing based on DRGs and, as a result, Romanian specialists became PCSI members over 10 years ago. The first DRG pilot projects in Romania occurred between 1996–1999, and in 2002 Romania officially introduced the DRG system.

The PCSI association and its annual conferences represented not only a “school” for Romanian specialists, but also a place to share local developments, successes, and problems encountered in the implementation of DRG in Romania. Now, however, it is time for Romania to share its recent experience of introducing its own DRG system – RO.vi.DRG – which began in 2010.

## Methods

The authors have done a comparative analysis between Romania and other countries which use, or are in the process of adopting, the DRG system. For both the Romanian situation and a comparison with other countries, the authors conducted a review of available literature. The authors also performed a quantitative analysis to highlight critical issues in system functioning and the impact of introducing Romanian classification.

## Results

The following is a list of the results obtained by the study.

1. Romania is a country with 10 years of experience in DRG utilization. Its health system is no longer in the beginning stages of DRG utilization. Romania’s new classification system is based on the AR DRG v.5 classification, and although some adaptations have been made for the Romanian situation, more still needs to be done.

2. Ongoing DRG system development and refinement activities require important resources. These resources are not only financial, but also human. Human resources, both at the central and hospital levels, are necessary to realize the next level of benefits from DRGs in Romania. A coherent, regular and strong training system is no longer just a requirement; it is an imperative necessity, not only for adequate financing, but also for the improvement and local adoption of the AR DRG classification system, so that it better reflects the Romanian hospital reality.

3. There are some prerequisites for obtaining correct results in hospital financing when using the DRG system. These are complete transparency of hospital-funds allocation, and the existence of a clear policy with defined objectives and long-term goals regarding hospital financing. The DRG system in Romania has currently been extended from 291 to 371 hospitals, but the total amount of money available for reimbursement still seems to be insufficient. As well, the reporting and financing system is not familiar enough to every hospital, and the benchmarking mechanism is insufficiently developed.

4. The experience of other countries where the DRG system works and produces good results shows that it is mandatory to have strong institutions involved in hospital-report monitoring. In addition, it is necessary to develop a clear set of regulations regarding the entire process of documentation, classification, coding, data processing and collection of patient-level clinical information.

5. As long as Romanian legislation considers the upgrading of a patient’s pathology in order to gain more funds just “an error” (which, in a worst-case situation, could lead to the return of the funds), up-coding will increase and create more dissatisfaction at the hospital-sector level. Starting in 2011, some analysis from National School of Public Health, Management and Professional Development in Health Bucharest (NSPHMPDHB) triggered controls of National Insurance House (NIH) at the hospital level. However, a concrete and planned mechanism for auditing coding is missing at the national level.

6. Continuous development of the DRG system is not merely a trend; it is a necessity. In order to have this development, it is essential to build effective communication pathways with hospitals in order to understand their reality, and to increase the capacity of the central institutions (NHIH, Ministry of Health, etc.) to design and respond to the new challenges.

7. Potential areas for development could be the following: emphasizing equitable hospital financing based on DRG; improving the accuracy of the patient classification system; improving the monitoring system; and increasing hospital efficiency.

## Conclusions

We could say that Romania started in the right direction by introducing and developing the DRG system. However, it is necessary to push for a stronger effort, and more professionalism and support, from the decision makers in order to not only keep the system working, but also to be sure of achieving the goals established at the moment of its implementation.

